# Reinterpretation of prostate cancer pathology by Appl1, Sortilin and Syndecan-1 biomarkers

**DOI:** 10.1038/s41597-024-03696-0

**Published:** 2024-08-08

**Authors:** Jessica M. Logan, Carmela Martini, Alexandra Sorvina, Ian R. D. Johnson, Robert D. Brooks, Maria C. Caruso, Chelsea Huzzell, Courtney R. Moore, Litsa Karageorgos, Lisa M. Butler, Prerna Tewari, Sarita Prabhakaran, Shane M. Hickey, Sonja Klebe, Hemamali Samaratunga, Brett Delahunt, Kim Moretti, John J. O’Leary, Douglas A. Brooks, Ben S.-Y. Ung

**Affiliations:** 1 Clinical and Health Sciences, University of South Australia, Bradley Building, City West Campus, North Terrace, Adelaide, SA 5000 Australia; 2 Centre for Cancer Biology, University of South Australia, North Terrace, Adelaide, SA 5000 Australia; 3 South Australian ImmunoGENomics Cancer Institute and Freemasons Centre for Male Health and Wellbeing, University of Adelaide, Adelaide, SA 5005 Australia; 4 Solid Tumour Program, Precision Cancer Medicine Theme, South Australian Health and Medical Research Institute, Adelaide, SA 5000 Australia; 5 Department of Histopathology, Trinity College Dublin, D02 PN40 Dublin, Ireland; 6 Department of Anatomical Pathology, College of Medicine and Public Health, Flinders University, Adelaide, SA 5042 Australia; 7 Aquesta Uropathology, The University of Queensland, Brisbane, QLD 4072 Australia; 8 Malaghan Institute of Medical Research, Wellington, 6012 New Zealand; 9 Discipline of Surgery, University of Adelaide, Adelaide, SA 5371 Australia; 10 Allied Health and Human Performance, University of South Australia, Adelaide, SA 5005 Australia; 11 Faculty of Medicine, Nursing and Health Sciences, Monash University, Melbourne, VIC 3800 Australia; 12 Quality Use of Medicines and Pharmacy Research Centre, University of South Australia City East Campus, Frome Rd, Adelaide, SA 5000 Australia

**Keywords:** Predictive markers, Tumour biomarkers

## Abstract

The diagnosis of prostate cancer using histopathology is reliant on the accurate interpretation of prostate tissue sections. Current standards rely on the assessment of Haematoxylin and Eosin (H&E) staining, which can be difficult to interpret and introduce inter-observer variability. Here, we present a digital pathology atlas and online resource of prostate cancer tissue micrographs for both H&E and the reinterpretation of samples using a novel set of three biomarkers as an interactive tool, where clinicians and scientists can explore high resolution histopathology from various case studies. The digital pathology prostate cancer atlas when used in conjunction with the biomarkers, will assist pathologists to accurately grade prostate cancer tissue samples.

## Background & Summary

Prostate cancer is the second most commonly diagnosed cancer in men with a global incidence of 1.41 million in 2020, which is expected to double by 2040^1^. The Gleason scoring system has been used for more than half a century to assess prostate cancer pathology and to inform on decisions for patient management and therapeutic intervention. The current International Society of Urological Pathology (ISUP) guidelines for the diagnosis of prostate cancer are based upon a modified Gleason grading system^2–8^, where morphology and architecture of malignant prostate glands are evaluated by routine Haematoxylin and Eosin (H&E) staining and histological examination. There are several challenges pathologists face when applying ISUP grading to prostate cancer on H&E-stained biopsy slides. Some challenges include (1) the distinction between the Gleason score 3 + 3 with tangential cutting artifacts vs. the Gleason score 3 + 4 with poorly-formed or fused glands, (2) the determining the relative amounts of pattern 3 and 4 (Gleason scores 3 + 4 vs. 4 + 3), and (4) the identification of a tertiary component of Gleason pattern 5^9–11^. Among these, differentiating ISUP 2 (3 + 4) versus ISUP 3 (4 + 3) is the most challenging due to disagreement of Gleason patterns 3 versus 4^9^. Unfortunately, these challenges are confounded by problems with complex morphological interpretation and inter-observer variability, which can lead to poor management and treatment selection for patients^8,12^. To transform prostate cancer pathology assessment, a new panel of biomarkers, consisting of Appl1, Sortilin and Syndecan-1 using immunohistochemistry (IHC), have been developed to accurately interpret the cancer biology, and for the first time assist in Gleason grading^13^. This new biomarker technology provides reliable detection of prostate cancer in patient tissue samples, and improved risk class stratification for ISUP grade grouping^13–15^. Other technologies are available including Ki67, p6 and P63 / AMACR cocktails however they are not utilised to assist in the assignment of ISUP grade groups. The recent introduction of these biomarkers into clinical practice as a laboratory developed test (LDT) in the United States necessitates training and reference resources to support the clinical utility of this new technology^16^.

**Fig. 1:**
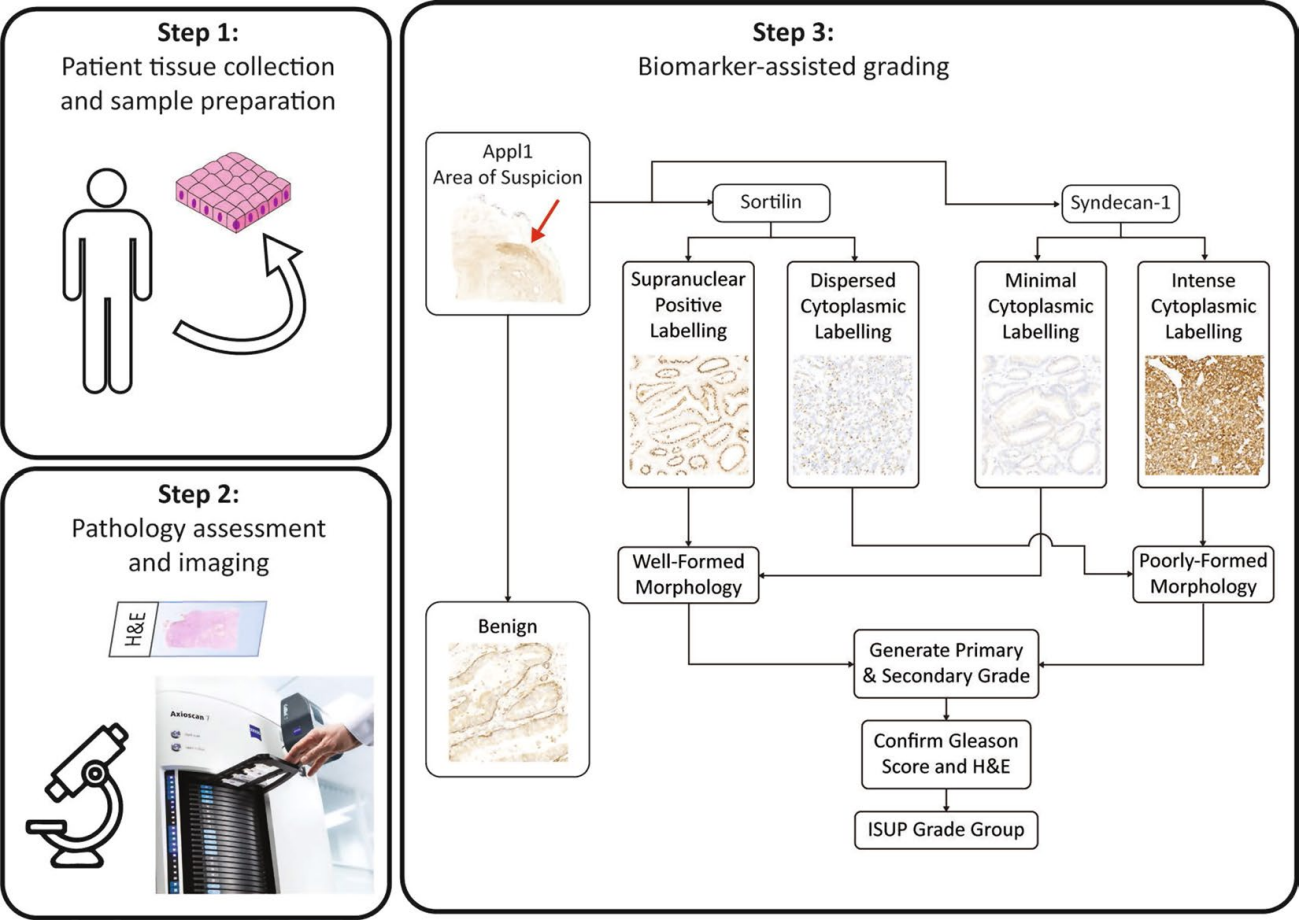
Workflow using the biomarker panel for IHC-assisted grading of prostate cancer tissue. During step 1, a section if examined with Appl1 labelling to identify areas of suspicion. The low power micrograph has an area towards the top right of the section (denoted in step 3 with a red arrow) which is moderately labelled (intensity 2+/ 3+) with Appl1 highlighting an area of suspicion glands which should be evaluated with Sortilin and Syndecan-1 as indicated in the workflow. For well-formed morphology, Sortilin labelling is moderate to high (intensity 2+/ 3+) and Syndecan-1 labelling is absent / low (intensity 0 / 1+). In poorly-formed morphology, Syndecan-1 labelling is moderate to high (intensity 2+/ 3+) and Sortilin labelling is absent / low (intensity 0 / 1+). Benign regions of the micrograph can be confirmed by moderate labelling of the basal cell layer with Appl1. Relative proportions of well-formed and poorly-formed malignant gland morphology can then be used to determine the appropriate ISUP Grade group.

A comprehensive resource of digitised Appl1, Sortilin and Syndecan-1 biomarker IHC labelled and routine H&E-stained prostate cancer slide images was compiled, together with clinical pathology annotations (Fig. 1) and were critical for their recent implementation into clinical practice. Here, we present a complete dataset of case studies showing grade groups from benign, and ISUP 1 to ISUP 5 with H&E stained tissue sections. This dataset is then reinterpreted together with our biomarkers to assist and improve the accuracy of the grading. These micrographs will ideally be used as a pathology reference system, an educational interpretation guide, and as an advanced training tool for biomarker-assisted ISUP grading. This method for more accurate and reliable Gleason grading was needed to improve the prognostic assessment in patients with prostate cancer, which will provide a more rigorous evidence-base for decisions on therapeutic intervention^5–8,12^. The underlying problems with Gleason grading include its reliance on morphology, which is often confounding or lacks sufficient detail to facilitate accurate interpretation of the complex pathology, and this is compounded by H&E staining which is not specific for the cancer^17,18^.

## Methods

### Sample acquisition

Prostate adenocarcinoma tissue sections were cut from de-identified Formalin Fixed Paraffin Embedded (FFPE) tissue samples, which were collected as part of routine diagnosis and were obtained either from the Australian Prostate Cancer Bioresource (APCB, Australia), Prostate Cancer Research Consortium (PCRC, Ireland), Flinders Medical Centre (Adelaide, South Australia) or Aquesta Uropathology (Brisbane, Queensland). Serial sections of tissue were either stained with routine H&E or Appl1, Sortilin or Syndecan-1 labelled by IHC as outlined previously^12^. Each tissue sample was then digitised in high magnification (40 × objective) using the Carl ZEISS AxioScan Z.1 and manually reviewed to ensure scan quality. A subset of prostate cancer digital micrographs have been previously published^13–15,19–22^, and can be used to supplement this digital resource. Basic clinical pathological data consisting of age, tumour stage, biochemical and clinical recurrence status were acquired from either the deidentified local electronic health or the bioresource records where available (see Ethics approval and consent to participate).

### Whole slide image processing

All image data was recorded in RGB (red, green and blue) colour format with a bit depth of 24-bit (8-bit per colour channel). Images were viewed using 0.45 gamma, with a maximum function applied, mimicking that of viewing slides under a physical microscope.

### Case study interpretation

**Fig. 2:**
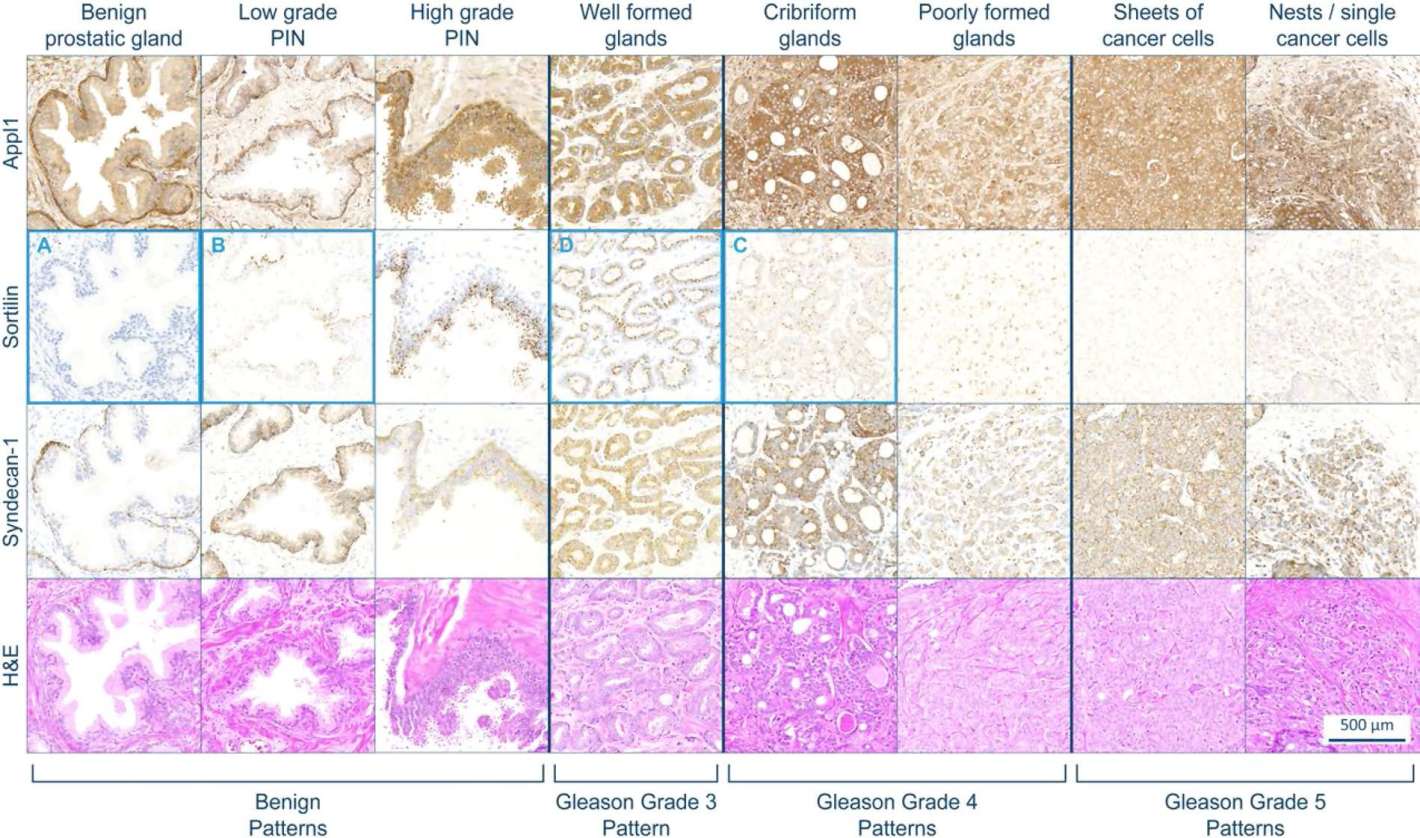
Gleason grade patterns comparing the biomarker panel with H&E. An example intensity scale is shown with Sortilin in row 2. No labelling is denoted with A (0+), low intensity labelling with B (1+), moderate intensity labelling with C (2+), and high intensity labelling with D (3+). A more comprehensive figure can be found in Martini *et al*.^13^.

The biomarkers Appl1, Sortilin and Syndecan-1 were selected for their unique pathological significance in prostate cancer and are designed to be used in a combinatorial assessment^13–15^, where specific examples can be found in the digital pathology prostate cancer atlas^23^ (10.5061/dryad.v9s4mw749) and are described in depth below. In prostate tissue, Appl1 is detected within basal cells of benign prostatic glands, often found within the nucleus of dysplastic cells in prostatic intraepithelial neoplasia (PIN), and has a cytoplasmic and diffuse labelling pattern within the malignant epithelial cells of the adenocarcinoma^13–15,22,24^. Clinically, Appl1 is intended to aid in confirming any suspicion of malignant prostatic glands identified during routine H&E screening and to identify regions of interest for grading when using the other two biomarkers, Sortilin and Syndecan-1. Specific examples of Appl1 labelling can be seen in the prostate cancer atlas within all presented cases^23^. IHC-assisted grading is performed using the biomarkers Sortilin and Syndecan-1; in PIN, Sortilin labelling is punctate and has a supranuclear distribution. In prostate adenocarcinoma, the labelling and distribution of Sortilin can be used to interpret differences between well-formed (intense granular and supranuclear location) and poorly-formed malignant glands (diffuse and disseminated labelling pattern, or absent). Syndecan-1 is detected within basal cells of benign prostatic glands and in malignant prostatic glands with poor-formed gland morphology. Syndecan-1 can also highlight inconspicuous lesions (e.g. small pockets of cancer) and single cancer cells with a differing intracellular distribution, compared to plasma cells (respectively cytoplasmic vs membranous labelling). Specific case study examples are outlined below and can be visualised from the Dryad data repository^23^. An example of this interpretation is illustrated in Fig. 2.

Benign Case Study: This case showed atrophy and seminal vesicles, but no cancerous glands were identified in the H&E stained section. Observing the serial sections using the biomarkers, the lack of cancerous glands was confirmed. It should be noted, that although this section is benign, distant blocks of the radical prostatectomy have cancerous glands present, and the patient was diagnosed with a tumour stage T3A. This patient did not biochemically relapse during the first 77 months post radical prostatectomy.

**Fig. 3:**
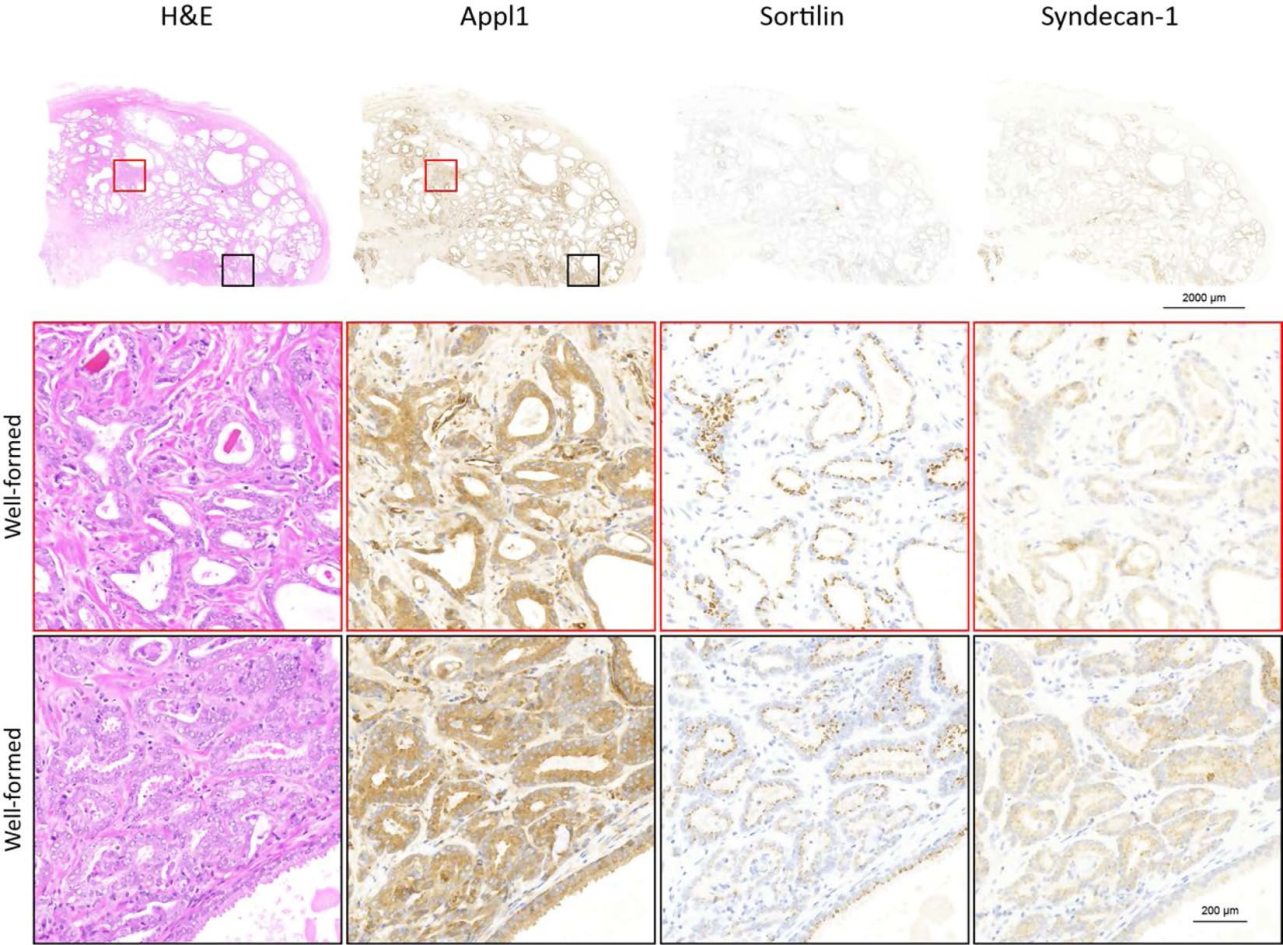
Patient with ISUP grade group 1 prostate adenocarcinoma. When evaluating the sample by H&E, the secondary pattern was initially graded as Gleason pattern. When re-evaluated with the biomarker panel this area indicates the secondary pattern is also well-formed glands. In evaluating Sortilin, all glands have polar punctate labelling supporting the interpretation of well-formed morphology. This interpretation was confirmed with minimal labelling of Syndecan-1. Representative images of the primary Gleason pattern is highlighted in red while the secondary Gleason pattern is highlighted in black.

ISUP 1 Case Study: For this case, H&E grading showed mainly well-formed glands with poorly-formed glands also present, leading to a grade group of ISUP 2. Reinterpretation using the biomarkers showed the case only presented well-formed glands, with no poorly-formed features, indicating grade group ISUP 1 (Fig. 3). The patient was diagnosed with a tumour stage T2C from a radical prostatectomy.

ISUP 2 Case Study: The H&E stained section for this case displayed both perineural and neural invasion, along with cribriform glands (poorly-formed gland morphology). Inflammatory infiltration was also present as well as glands with glomeruloid features. Upon reinterpretation with the biomarkers, these features were confirmed, both Sortilin and Syndecan-1 labelling intensity was high in the cancerous regions, indicating a grade grouping of ISUP 2. The patient had extracapsular extension and extensive neural involvement and did not biochemically relapse during the first 107 months post radical prostatectomy.

**Fig. 4:**
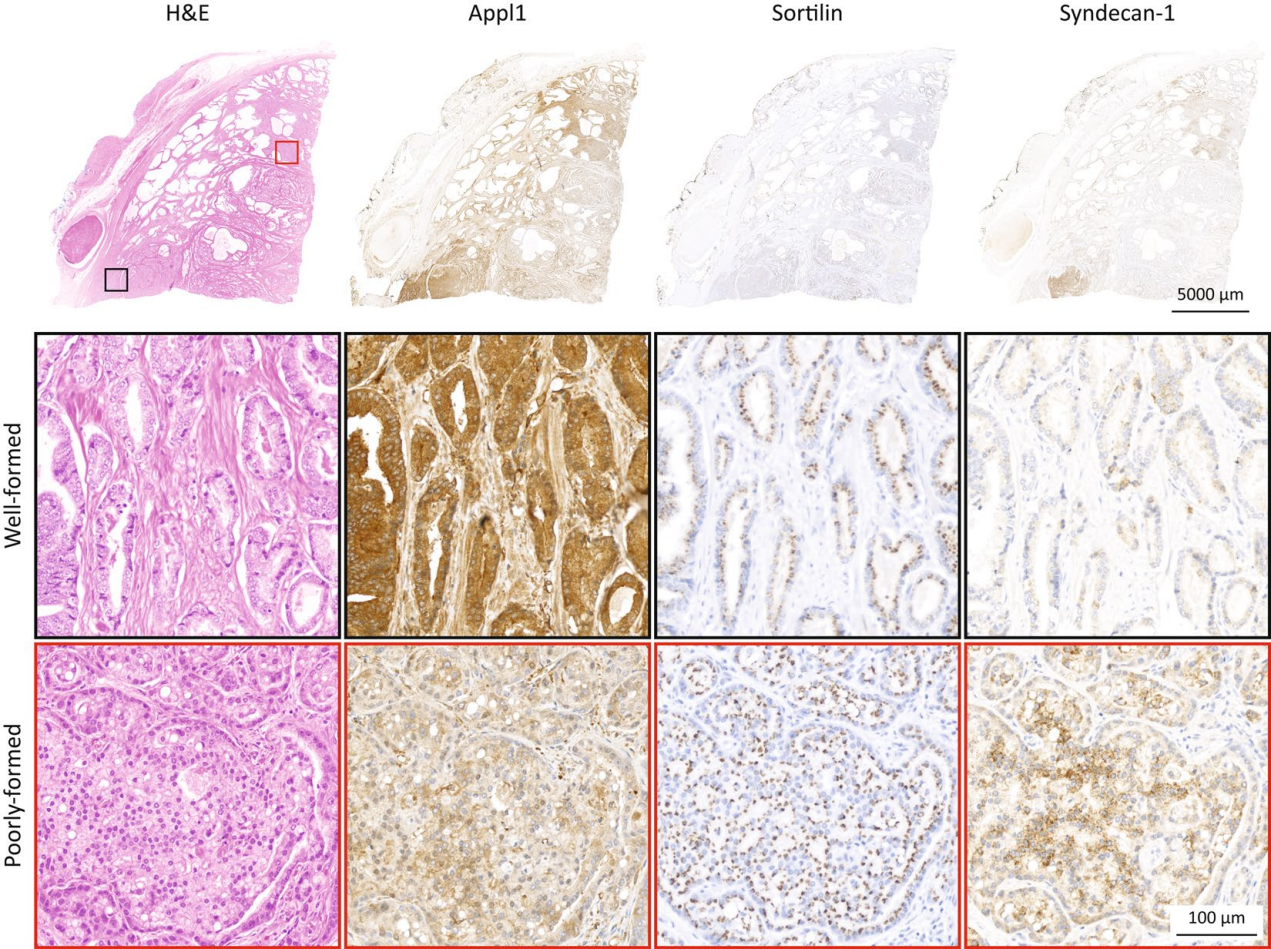
Patient with ISUP grade group 3 prostate adenocarcinoma. Top row; H&E staining and the biomarkers, Appl1, Sortilin and Syndecan-1 in radical prostatectomies. Middle and bottom rows; examples of well-formed and poorly-formed gland are shown respectively in black and red squares. The biomarkers assist with grading, showing a difference in intensity and clearly highlighting the cancer foci. Using H&E, this sample was originally graded as ISUP 2 however when reassessed using the biomarker panel the sample was increased to ISUP 3. In the bottom left of the micrograph, the black box highlights a region of well-formed malignant glands. Sortilin labelling is moderate (intensity 2+) while Syndecan-1 labelling is low (intensity 1+). Comparatively, in the red box the cribriform gland is also moderately labelled with Sortilin (intensity 2+) but Syndecan-1 intensity has increased also (intensity 2+). When reviewing the micrograph, Syndecan-1 labelling >2+ intensity (moderate - high) is evident in multiple areas indicating a greater predominance of Gleason pattern 4 glands hence the grade group change.

ISUP 3 Case Study: This case showed perineural invasion, with poorly-formed glands forming nests in the H&E stained section, with the tumour infiltrating extra prostatic adipose tissue, leading to an assessment grade group of ISUP 3. When observing with the biomarkers, these features were confirmed, showing a greater portion of poorly-formed glands over well-formed glands, reconciling the initial grade group of ISUP 3 (Fig. 4). The patient had a tumour stage T2C and did not biochemically relapse nor clinically recur during the first 125 months post radical prostatectomy.

**Fig. 5:**
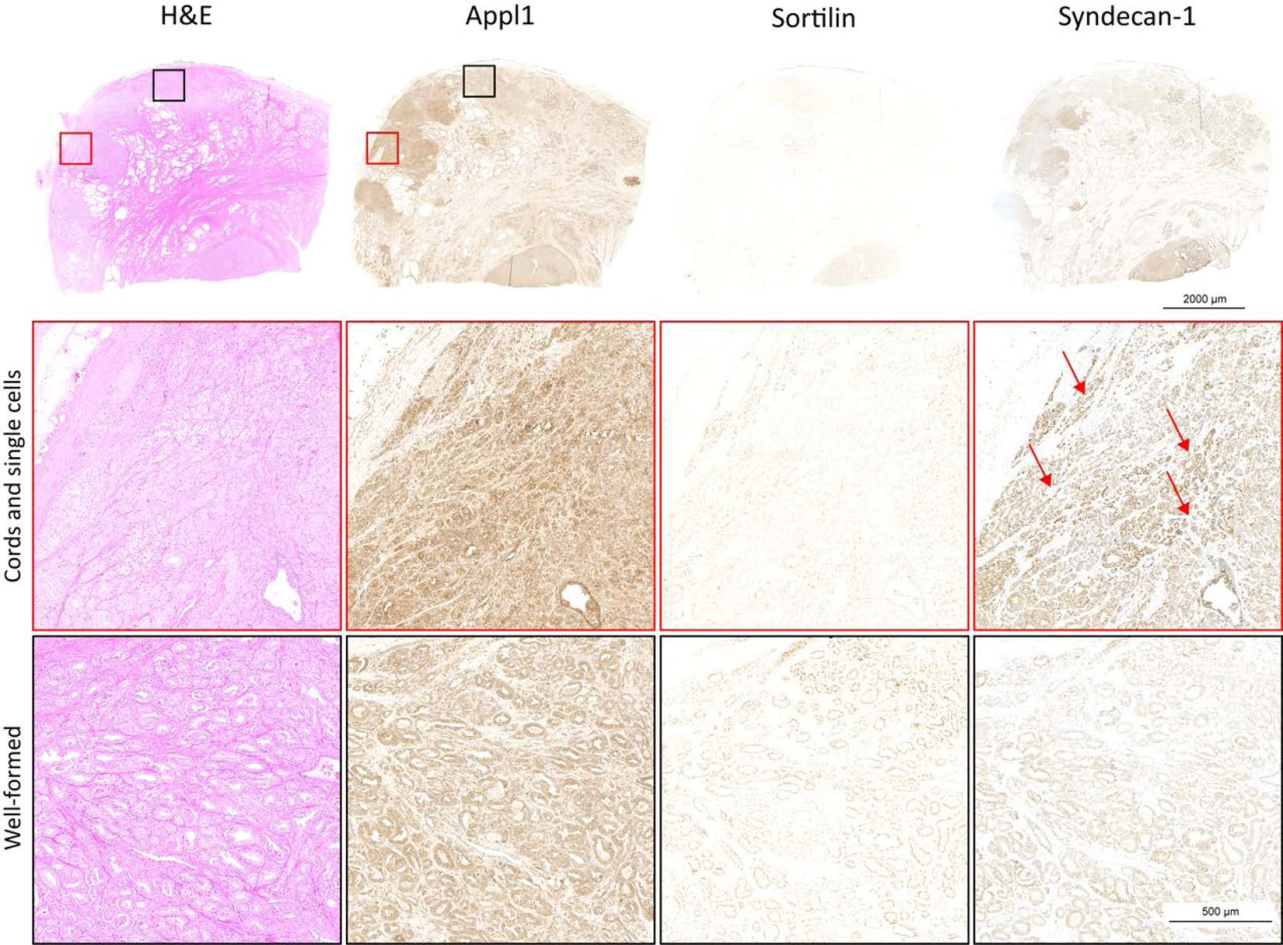
Patient with ISUP grade group 4 prostate adenocarcinoma. H&E illustrates a primary grade of Gleason pattern 4 with a majority of poorly-formed glands and a minor grade of Gleason pattern 3 with well-formed glands giving rise to an ISUP grade group of 3. Reassessment with the biomarkers highlighted the presence of nests, cords and single cells throughout the poorly-formed regions to increase the Gleason pattern identified from 4 to 5 in the primary region of the tumour. Syndecan-1 labelling is strong and highlights nests, cords and single cells (indicated by the red arrow) while Sortilin has lost polarity and has minimal labelling (<1+ intensity) as illustrated in the higher power micrographs. This sample was regraded to ISUP 4 (Gleason grade 5 + 3) from the original ISUP 3 by H&E.

ISUP 4 Case Study: In this case study, the H&E section showed the adenocarcinoma infiltrating extra prostatic adipose and fibrous tissue, with perineural invasion, along with poorly-formed glands. Adjacent sections labelled with the biomarkers showed single cells and cords were present (Gleason pattern 5) and confirmed perineural invasion along with perivascular invasion. The additional single cell and cord features as well as the predominant poorly-formed morphology indicated grade group ISUP 4 (Gleason grade 5 + 3) over the initial H&E grading of ISUP 3 (Gleason grade 4 + 3) (Fig. 5). This prostate tissue sample was obtained from a patient who had undergone a radical prostatectomy and had disease that was originally assessed, based on pathological examination, as tumour stage T3B (with extension of tumour into the seminal vesicles) without nodal involvement. The patient had developed non-metastatic biochemical relapse in 23 months and, following radiation therapy, had clinical recurrence of prostate cancer in 53 months after initial diagnosis. Prostatectomy findings confirmed that the patient had high-grade cancer.

ISUP 5 Case Study: The H&E stained section displayed both perineural and perivascular invasion, single cells and sheets, with intraductal carcinoma present. With the biomarker labelled sections, these features were also confirmed. As with the ISUP 4 case above, the intensity levels of Sortilin were low, while Syndecan-1 intensity was high. This prostate tissue sample was obtained from a patient who had undergone a radical prostatectomy and had disease that was originally assessed, based on pathological examination, as tumour stage T3B (with extension of tumour into the left seminal vesicles) without nodal involvement. The patient had developed non-metastatic biochemical relapse in 2.7 months and, following radiation therapy, had clinical recurrence of prostate cancer in 25 months after initial diagnosis. Prostatectomy findings confirmed that the patient had ISUP grade 5 prostate cancer.

### Biomarker utility

**Fig. 6:**
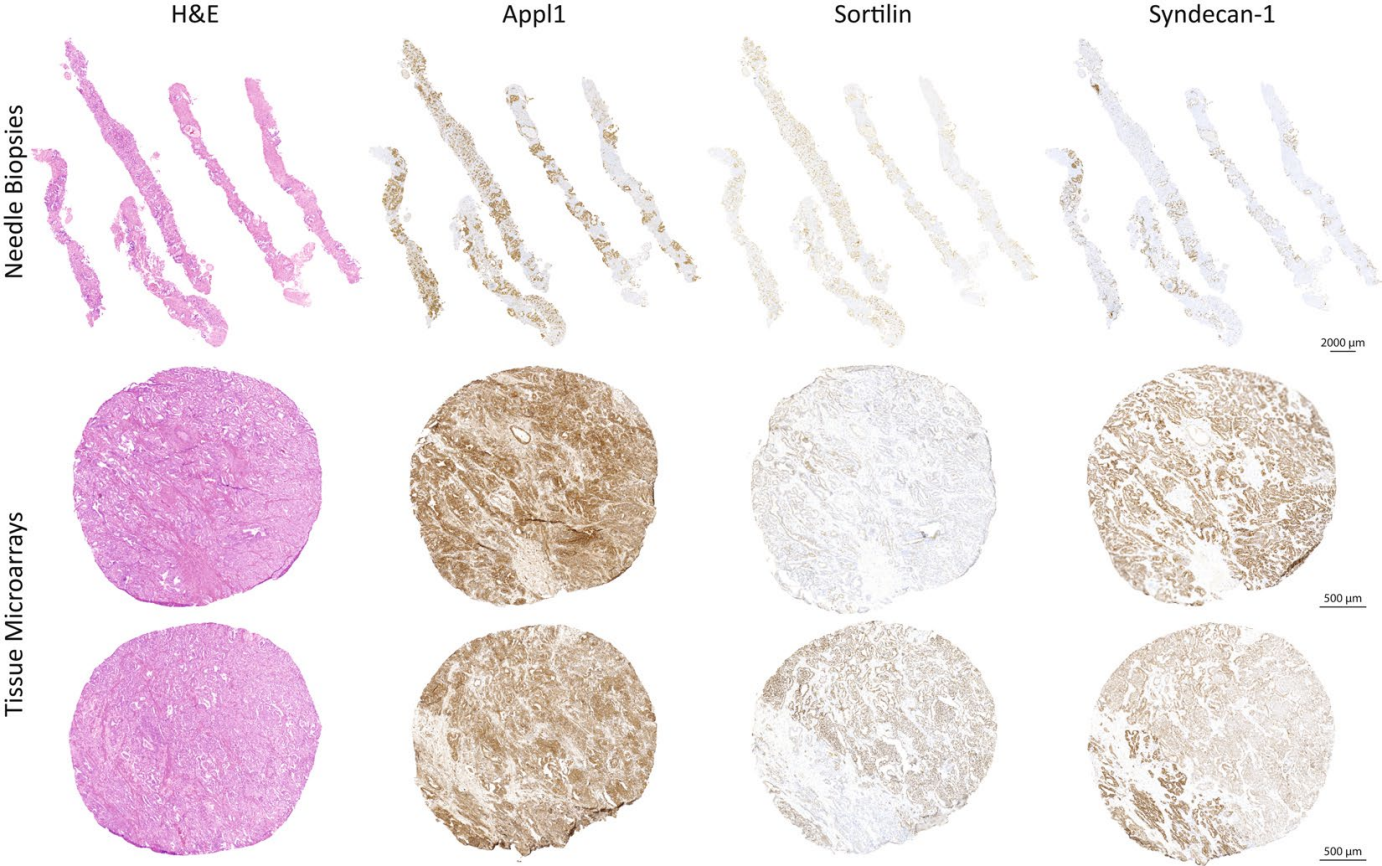
The biomarker panel allows for flexible utility in multiple tissue section types, including needle biopsies (top row), and TMAs (bottom two rows). Tumour foci are clearly highlighted, and the variations in intensity in Sortilin and Syndecan-1 can assist with grading. The needle biopsy sample was initially graded as ISUP 1 using H&E and was later confirmed to be ISUP 1 using the biomarker panel. Within each core, Appl1 labelling identified both benign and suspicious glands. These glands when assessed with Sortilin and Syndecan-1 and confirmed to have well-formed morphology consistent with ISUP 1. The TMA was originally graded as ISUP 3 by H&E. The first core contains both well-formed and poorly-formed malignant glands, however the proportion of poorly-formed morphology is larger than well-formed. Similarly, poorly-formed glands specifically cribriform glands predominated with smaller regions of well-formed malignant glands correlating with the Sortilin and Syndecan-1 labelling of the cores and an ISUP 3 grade group. No benign glands were evident in either the H&E stained or IHC labelled TMA cores.

Figure 6 and the digital pathology prostate cancer atlas^23^ present digital micrographs of the biomarker labelling in prostatectomy tissue samples and exemplifies the clinical utility in confirming the cancer pathology and grading after surgical ressection^13,14^. Other types of tissue samples, such as needle biopsies, tissue micro arrays (TMAs), transurethral resection of the prostate (TURPs) blocks (e.g. needle cores and microarrays in Fig. 6), may also be assessed using these biomarkers, and offer significant advantages in clinical practice^25–28^. This biomarker panel enables accurate and consistent grading of tissue samples, reducing inter-observer variability^13–15^, to make more precise diagnoses and treatment decisions. The biomarkers are easily adapted to different tissue processing protocols employing well established IHC techniques, thus enabling their utility on different technology platforms (Ventana, Omnis and Agilent platform technologies). The atlas and online resource highlight many cases where histopathology assessment using H&E in isolation can be challenging using morphology alone, and the biomarkers enable higher clarity and objectivity to assist with grading (see Dryad data repository, cases benign, and ISUP grade groups 1 to 5^23^). The digital pathology prostate cancer atlas is a comprehensive resource supported by an online database which allows for enhanced learning by comparing observations made using traditional H&E staining and biomarker guided technology, minimising problems with inter-observer variation.

### Imaging

For the digital pathology prostate cancer atlas (and Figures), whole slides of H&E stained or Appl1, Sortilin and Syndecan-1 labelled serial tissue sections were imaged using a digital slide scanner (Carl ZEISS AxioScan Z.1), at 40× magnification (Carl Zeiss Plan-Apochromat 40× NA 0.95) in brightfield. This magnification provided a good trade-off between acquisition speed, image data size and morphological resolution. All images were captured using the same scanning profile to ensure that the white balance, light illumination intensity and exposure times were identically acquired for each set of slides.

## Data Records

All images were recorded using the Carl Zeiss Image format (*.CZI) using ZEISS Zen Blue software (version 3.7, Carl Zeiss GmbH, Jena, Germany) with the supplied acquisition workstation computer. Viewing and editing of images was also performed using Zen Blue software. All images, file names and metadata were de-identified from patient data, and a unique identifier was assigned to each image set, maintaining data integrity and separation. Patient outcome data was recorded in a secure password-protected and user access-restricted Microsoft Excel spreadsheet document (version 2302, Redmond, USA).

All digital micrographs used in the presented case studies are freely available via the online repository Dryrad^23^, along with the digital pathology prostate cancer atlas and an instructional video on how to fully utilise and interact with the atlas. Sample interpretation data from a panel of medical scientists is also available in comma-separated value (*.CSV) format for all the presented case studies.

## Technical Validation

All cases were initially selected based upon the health records held directly by the bioresource manager or through the authorized custodian of the tissue samples. To ensure conformity with the Royal College of Pathologists of Australasia (RCPA) prostate cancer assessment guidelines (reported within the American Joint Committee on Cancer (AJCC) 2018 and guidelines updated by ISUP in the congress meeting of 2018), tissue sections were reviewed at two time points by independent clinical pathologists who were experienced in genitourinary cancers and focused particularly on prostate cancer. The first review was conducted by the pathologist associated with each bioresource, before sample inclusion into the study. Upon receipt of the samples for the research program, a tumour board (*n* = 11, chaired by J.J.O’L) also reviewed the pathology within each patient sample using routine pathology techniques before several pathologists (*n* = 6; Australia, New Zealand and Ireland), scientists (*n* = 4) and the tumour board (*n* = 11) assessed the same samples using the biomarkers Appl1, Sortilin and Syndecan-1^13,14^.

## Usage Notes

**Fig. 7:**
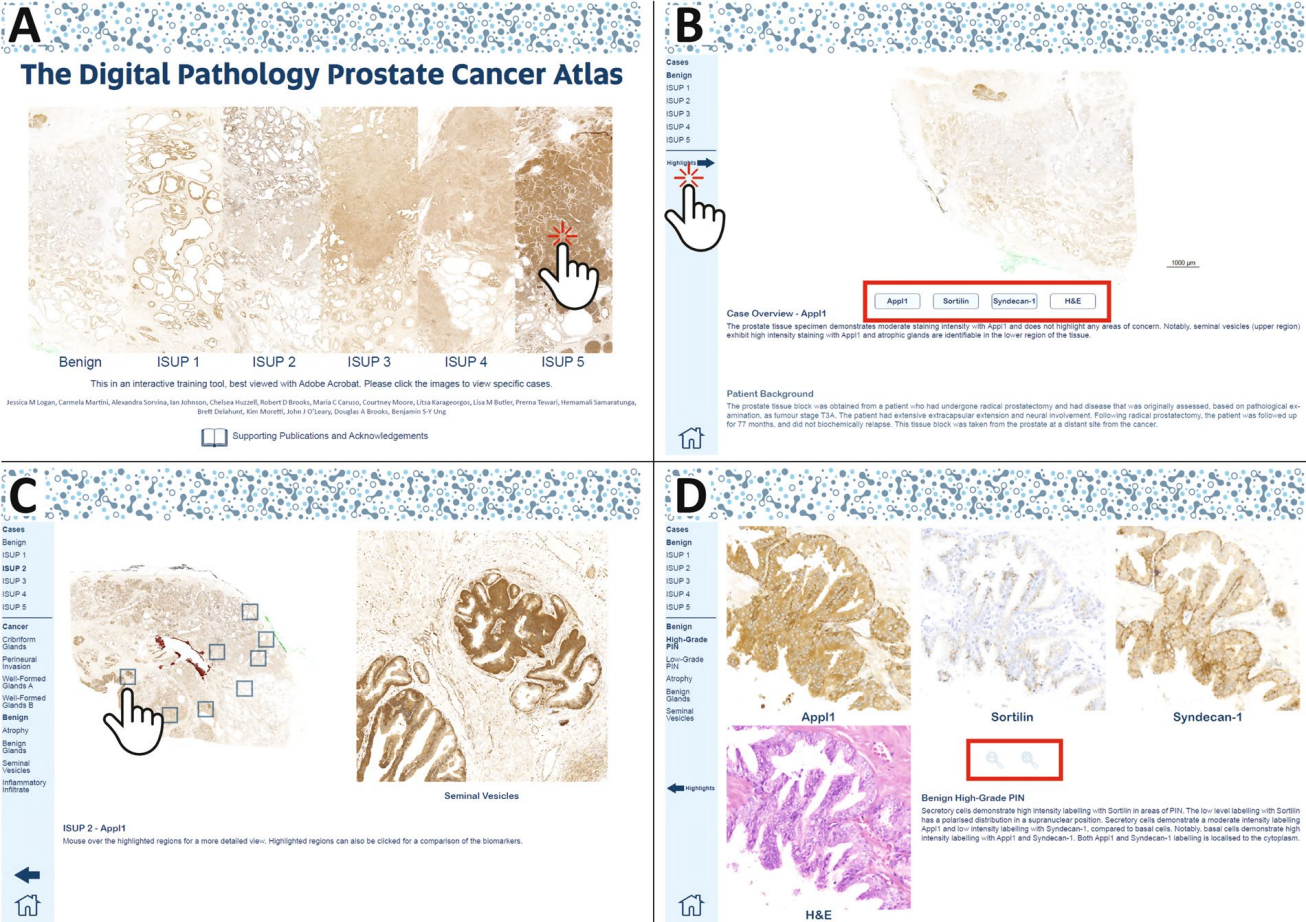
The Digital Pathology Prostate Cancer Atlas is an interactive easy-to-use PDF document containing representative prostatectomy images. (**A**) The title page enables users to select case studies ranging from benign to ISUP grade group 5, along with a link to supporting documentation at the bottom-center of the page. (**B**) The overview page of a case study enables users to navigate serial sections labelled with the panel of biomarkers, and H&E staining by hovering or clicking the buttons highlighted inside the red box. The highlights page or other case studies can be accessed by clicking the links on the left-hand pane. (**C**) The highlights page enables users to hover a cursor over specific highlights outlined on the tissue section, with a magnified view shown on the right-hand side. Clicking the specific highlights on the left-hand pane or the highlight squares will navigate to the chosen highlight. (**D**) A specific highlight page enables users to zoom in or out of the selected hightlight by clicking the zoom in/out buttons shown in the red box, or navigate using the left-hand pane.

Our specially developed digital pathology prostate cancer atlas comprised of micrographs from the APCB cohort is designed to be user interactive, highlighting key cancer features for H&E and IHC-assisted ISUP grade group assessment; hence is presented as an interactive portable document format (PDF) and online resource. The digital pathology prostate cancer atlas is best viewed using Adobe Acrobat Reader software (Adobe Inc, San Jose, USA) to make full use of the PDF document’s functionality. The atlas begins with a front page, where users can select and click to access representative images of benign through to ISUP grade group 5 cases of prostate cancer. The supporting material for the atlas can be accessed at the bottom of the front page with a button linking to the back matter (Fig. 7A)^23^. After selecting a case, the overview page of the case will be shown along with de-identified information on the patient background. The user can then hover a cursor over the biomarkers Appl1, Sortilin and Syndecan-1 or the H&E buttons to view the respective staining or IHC biomarker labelling of the prostatectomy tissue samples (Fig. 7B). On the left-hand panel, the highlights button can be clicked, along with links to other cases and the home button to bring the user back to the front page. Moving to the highlights page, users can hover the cursor over specific cancer features listed on the left-hand panel, or over the prostatectomy to view a magnified image (Fig. 7C). Clicking on either the feature name on the left-hand panel or the highlighted square on the prostatectomy will bring the user to the selected highlights page, showing the user a more detailed higher magnification view of the cancer feature. A zoom in or zoom out button is centrally located on the feature page, and users can see lower or higher-powered magnifications of the selected feature (Fig. 7D).

The novel biomarkers, Appl1, Sortilin and Syndecan-1 have been proven to assist with H&E diagnosis, with improved grading and prognostic prediction accuracy of patient outcomes^13–15,22^. In addition, the biomarkers are able to assist with more accurate determination of specific histopathology, such as intraductal carcinoma of the prostate^15^ and high-grade prostatic intraepithelial neoplasia^22^. The biomarkers have been developed as an LDT, and are now currently used in clinical practise in the United States of America^16^. The digital pathology prostate cancer atlas acts as an interactive tool to help educate and assist clinicians to better diagnose patients with prostate cancer and achieve improved outcomes.

The biomarker technology is amenable to a digital pathology Artificial Intelligence (AI) solution to further remove subjective inter-observer variability and to assess specific aspects of prostate cancer pathology. Preliminary algorithms to improve the efficacy of an AI solution are already under development and algorithms will be integrated with machine learning to produce a precision medicine solution for prostate cancer tissue pathology assessment.

The digital pathology prostate cancer atlas was developed using Adobe InDesign (Adobe Inc, San Jose, USA). There was no custom code generated for this manuscript and is therefore not available. The digital micrographs can be opened using any imaging software able to open the BigTIFF image format.

### Ethics approval and consent to participate

Approval was obtained from the Human Research Ethics Committee of the University of South Australia (Application IDs: 201907 and 36070). Patients with prostate cancer were accessed from several bioresources where their custodians, obtained informed consent to provide their samples to the bioresource.

## References

[1] World Health Organisation. at www.who.int (2023).

[2] Gleason, D. F. Classification of prostatic carcinomas. *Cancer Chemother. Reports* **50**, 125–8 (1966). 5948714

[3] Samaratunga, H. *et al*. The prognostic significance of the 2014 International Society of Urological Pathology (ISUP) grading system for prostate cancer. *Pathology* **47**, 515–519 (2015). 26325670 10.1097/PAT.0000000000000315

[4] Gleason, D. F. & Mellinger, G. T. Prediction of Prognosis for Prostatic Adenocarcinoma by Combined Histological Grading and Clinical Staging. *J. Urol.* **111**, 58–64 (1974). 4813554 10.1016/S0022-5347(17)59889-4

[5] Epstein, J. I. *et al*. The 2014 International Society of Urological Pathology (ISUP) Consensus Conference on Gleason Grading of Prostatic Carcinoma. *Am. J. Surg. Pathol.* **40**, 244–252 (2016). 26492179 10.1097/PAS.0000000000000530

[6] Epstein, J. I., Allsbrook, W. C., Amin, M. B. & Egevad, L. L. The 2005 International Society of Urological Pathology (ISUP) Consensus Conference on Gleason Grading of Prostatic Carcinoma. *Am. J. Surg. Pathol.* **29**, 1228–1242 (2005). 16096414 10.1097/01.pas.0000173646.99337.b1

[7] van Leenders, G. J. L. H. *et al*. The 2019 International Society of Urological Pathology (ISUP) Consensus Conference on Grading of Prostatic Carcinoma. *Am. J. Surg. Pathol.* **44**, e87–e99 (2020). 32459716 10.1097/PAS.0000000000001497 PMC7382533

[8] Srigley, J. R. *et al*. Controversial issues in Gleason and International Society of Urological Pathology (ISUP) prostate cancer grading: proposed recommendations for international implementation. *Pathology* **51**, 463–473 (2019). 31279442 10.1016/j.pathol.2019.05.001

[9] Egevad, L. *et al*. Identification of areas of grading difficulties in prostate cancer and comparison with artificial intelligence assisted grading. *Virchows Arch.* **477**, 777–786 (2020). 32542445 10.1007/s00428-020-02858-w PMC7683442

[10] Hassan, O. & Matoso, A. Clinical significance of subtypes of Gleason pattern 4 prostate cancer. *Transl. Androl. Urol.* **7**, S477–S483 (2018). 30363452 10.21037/tau.2018.02.06 PMC6178320

[11] Flood, T. A. & Schieda, N. Beyond the Gleason score: the prognostic significance of prostate cancer subtypes. *Transl. Androl. Urol.* **7**, S260–S261 (2018). 29932176 10.21037/tau.2018.04.01 PMC5989107

[12] Goodman, M. *et al*. Frequency and determinants of disagreement and error in gleason scores: A population-based study of prostate cancer. *Prostate* **72**, 1389–1398 (2012). 22228120 10.1002/pros.22484 PMC3339279

[13] Martini, C. *et al*. Aberrant protein expression of Appl1, Sortilin and Syndecan-1 during the biological progression of prostate cancer. *Pathology* **55**, 40–51 (2023). 36089417 10.1016/j.pathol.2022.08.001

[14] Logan, J. M. *et al*. Prediction of Prostate Cancer Biochemical and Clinical Recurrence Is Improved by IHC-Assisted Grading Using Appl1, Sortilin and Syndecan-1. *Cancers (Basel).* **15**, 3215 (2023). 37370825 10.3390/cancers15123215 PMC10296524

[15] Sorvina, A. *et al*. Appl1, Sortilin and Syndecan-1 immunohistochemistry on intraductal carcinoma of the prostate provides evidence of retrograde spread. *Pathology*, 10.1016/j.pathol.2023.05.004 (2023). 10.1016/j.pathol.2023.05.004 37422404

[16] Quest Diagnostics Launches Novel Prostate Cancer Test Aimed at Improving Diagnosis and Grading. at https://newsroom.questdiagnostics.com/2023-07-13-Quest-Diagnostics-Launches-Novel-Prostate-Cancer-Test-Aimed-at-Improving-Diagnosis-and-Grading (2023).

[17] Srigley, J. R. Benign mimickers of prostatic adenocarcinoma. *Mod. Pathol.* **17**, 328–348 (2004). 14976539 10.1038/modpathol.3800055

[18] Egevad, L. *et al*. Benign mimics of prostate cancer. *Pathology* **53**, 26–35 (2021). 33070957 10.1016/j.pathol.2020.08.006

[19] Johnson, I. R. D. *et al*. Endosomal gene expression: a new indicator for prostate cancer patient prognosis? *Oncotarget* **6**, 37919–37929 (2015). 26473288 10.18632/oncotarget.6114 PMC4741974

[20] Johnson, I. R., Parkinson-Lawrence, E. J., Butler, L. M. & Brooks, D. A. Prostate cell lines as models for biomarker discovery: Performance of current markers and the search for new biomarkers. *Prostate* **74**, 547–560 (2014). 24435746 10.1002/pros.22777

[21] Johnson, I. R. D. *et al*. Altered endosome biogenesis in prostate cancer has biomarker potential. *Mol. Cancer Res.* **12**, 1851–1862 (2014). 25080433 10.1158/1541-7786.MCR-14-0074 PMC4757910

[22] Martini, C. *et al*. Distinct patterns of biomarker expression for atypical intraductal proliferations in prostate cancer. *Virchows Arch*. 10.1007/s00428-023-03643-1 (2023). 10.1007/s00428-023-03643-1 PMC11522086 37704825

[23] Logan, J. M. *et al*. Reinterpretation of prostate cancer pathology by Appl1, Sortilin and Syndecan-1 biomarkers. *Dryad*10.5061/dryad.v9s4mw749 (2024). 10.5061/dryad.v9s4mw749 PMC11310308 39117701

[24] Lazniewska, J. *et al*. Dynamic interplay between sortilin and syndecan-1 contributes to prostate cancer progression. *Sci. Rep.* **13**, 13489 (2023). 37596305 10.1038/s41598-023-40347-7 PMC10439187

[25] Jhavar, S. *et al*. Construction of tissue microarrays from prostate needle biopsy specimens. *Br. J. Cancer* **93**, 478–482 (2005). 16091762 10.1038/sj.bjc.6602726 PMC2361582

[26] Singh, S. S. *et al*. Feasibility of constructing tissue microarrays from diagnostic prostate biopsies. *Prostate* **67**, 1011–1018 (2007). 17476688 10.1002/pros.20603

[27] McCarthy, F. *et al*. An improved method for constructing tissue microarrays from prostate needle biopsy specimens. *J. Clin. Pathol.* **62**, 694–698 (2009). 19638540 10.1136/jcp.2009.065201 PMC2709943

[28] Vogel, U. Overview on Techniques to Construct Tissue Arrays with Special Emphasis on Tissue Microarrays. *Microarrays* **3**, 103–136 (2014). 27600339 10.3390/microarrays3020103 PMC5003444

